# Supplementary material for: Loss of the APP regulator RHBDL4 preserves memory in an Alzheimer’s disease mouse model
Source: Cell Death Dis. 2025 Apr 12;16(1):280. doi: 10.1038/s41419-025-07579-z (PMC11993729; doi:10.1038/s41419-025-07579-z)
Supplement: Supplementary file 1 — Supplmentary Figure 1 [file 41419_2025_7579_MOESM1_ESM.pdf]

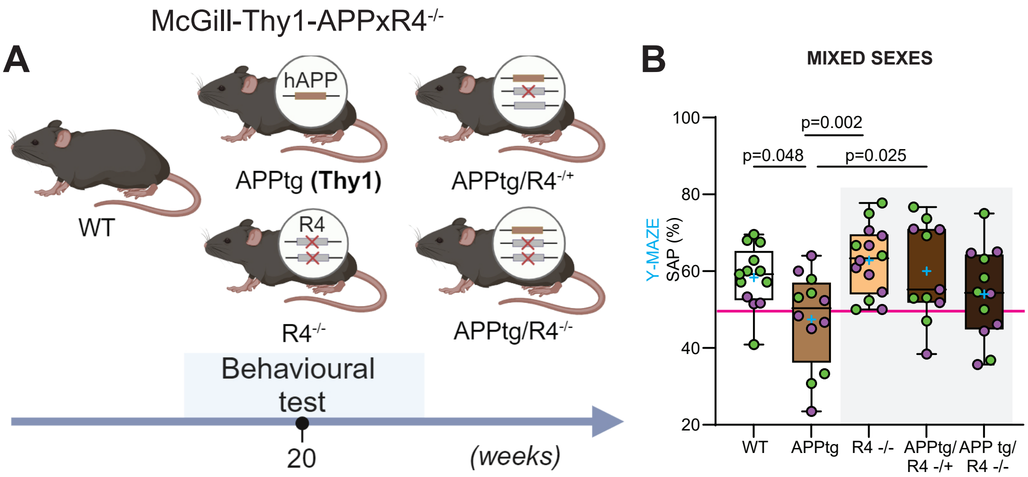

Supplemental Figure 1: Cognitive defects are improved in the absence of RHBDL4 expression in a different APPtg model.

A. Schematic representation of experimental design and analysis timeline for preliminary cohort of APPtg McGill-Thy1-APP mice crossed to the $R4^{-/-}$ model.

B. Spontaneous alternation performance (SAP) score from Y maze test of WT, APPtg, $R4^{-/-}$, APPtg/$R4^{-/+}$, and APPtg/$R4^{-/-}$ mice. Female data points are in purple and male in green, n=12-14 per group. Box and whisker plots represent minimum to maximum values with median center lines while blue "+" represents the mean. One-way ANOVA (p=0.008) with Dunnett's multiple comparison test, significant p-values for post hoc analysis reported. Assumptions of normality and variance were verified using Shapiro-Wilk test and Brown-Forsythe test, respectively.
