# Supplementary material for: Loss of the APP regulator RHBDL4 preserves memory in an Alzheimer’s disease mouse model
Source: Cell Death Dis. 2025 Apr 12;16(1):280. doi: 10.1038/s41419-025-07579-z (PMC11993729; doi:10.1038/s41419-025-07579-z)
Supplement: Supplementary file 2 — Raw Western blot data [file 41419_2025_7579_MOESM2_ESM.pdf]

FIGURE 1C

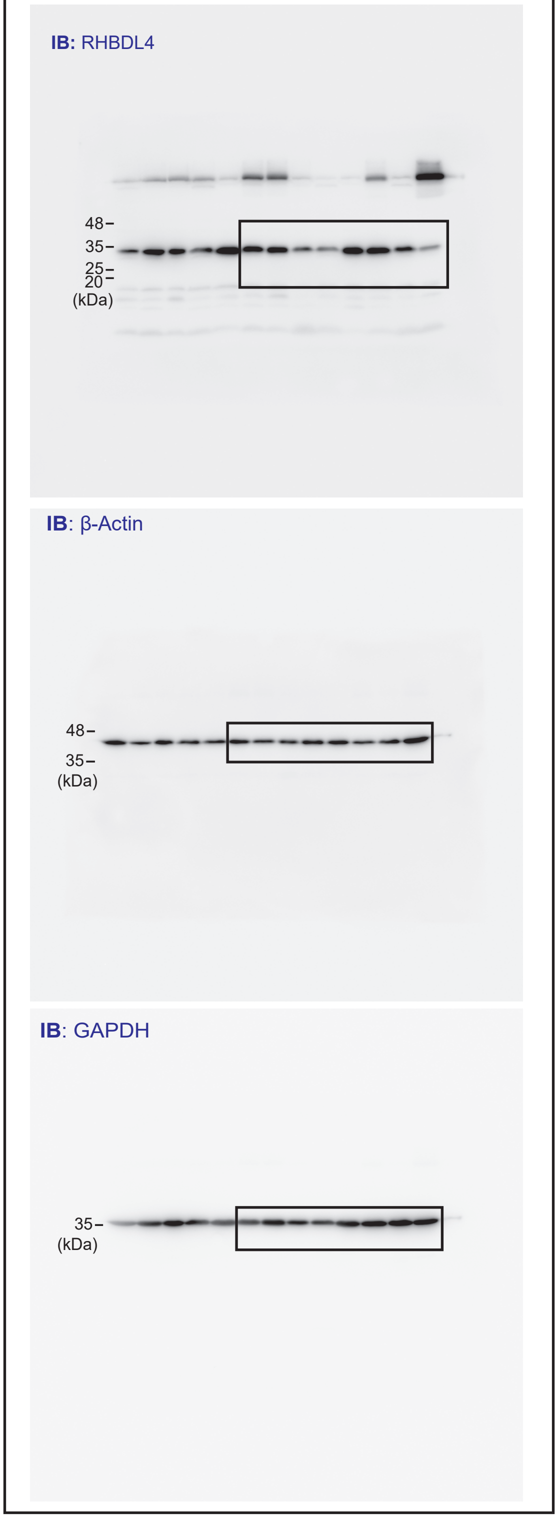

FIGURE 1E

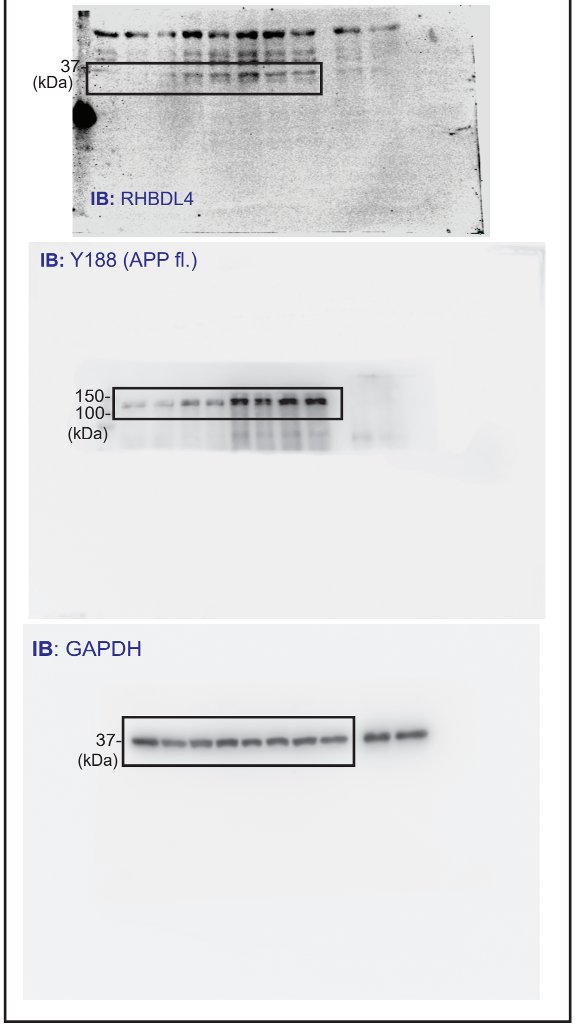

FIGURE 2B

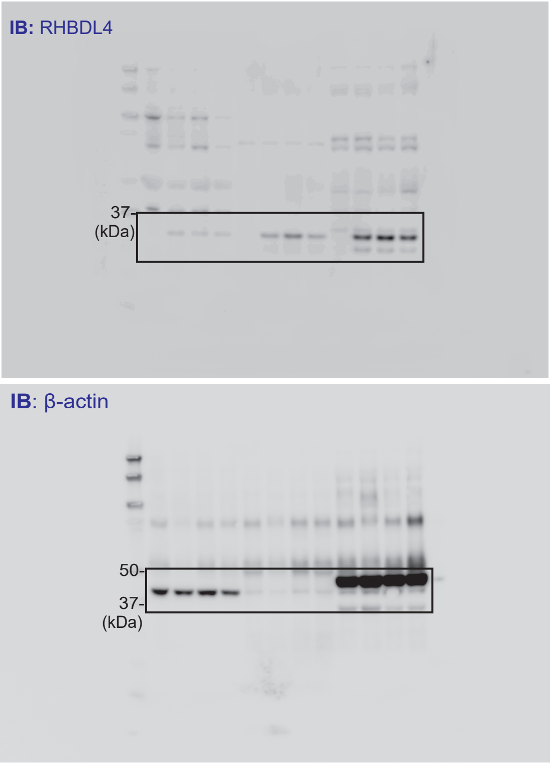

FIGURE 2D

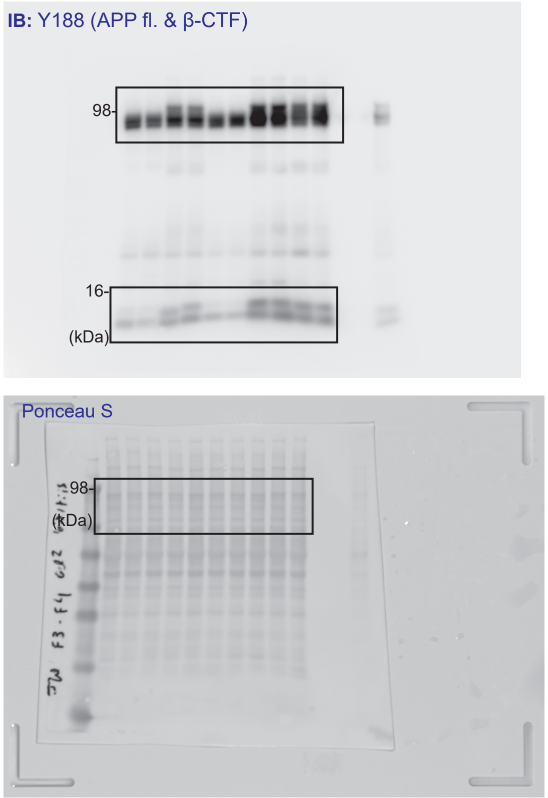

FIGURE 2C

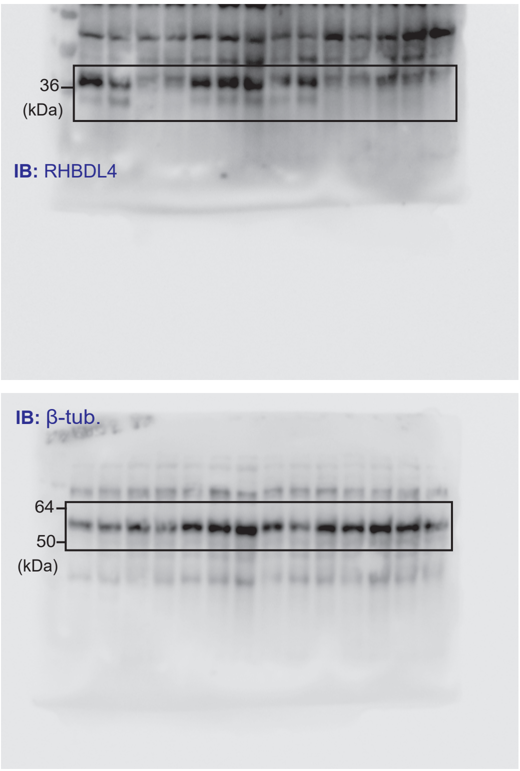

FIGURE 4D

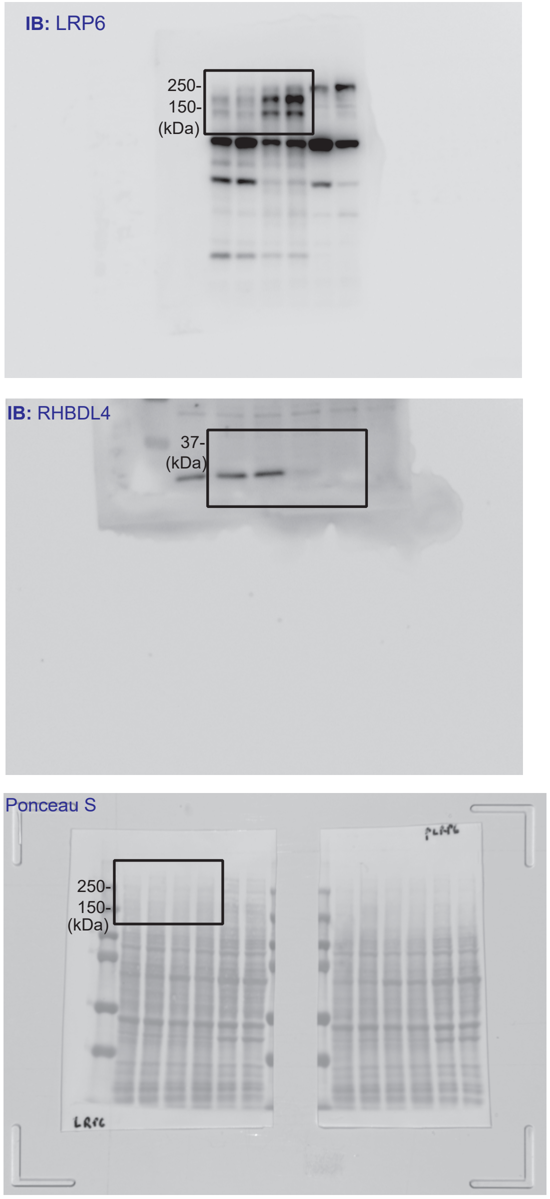

FIGURE 5A

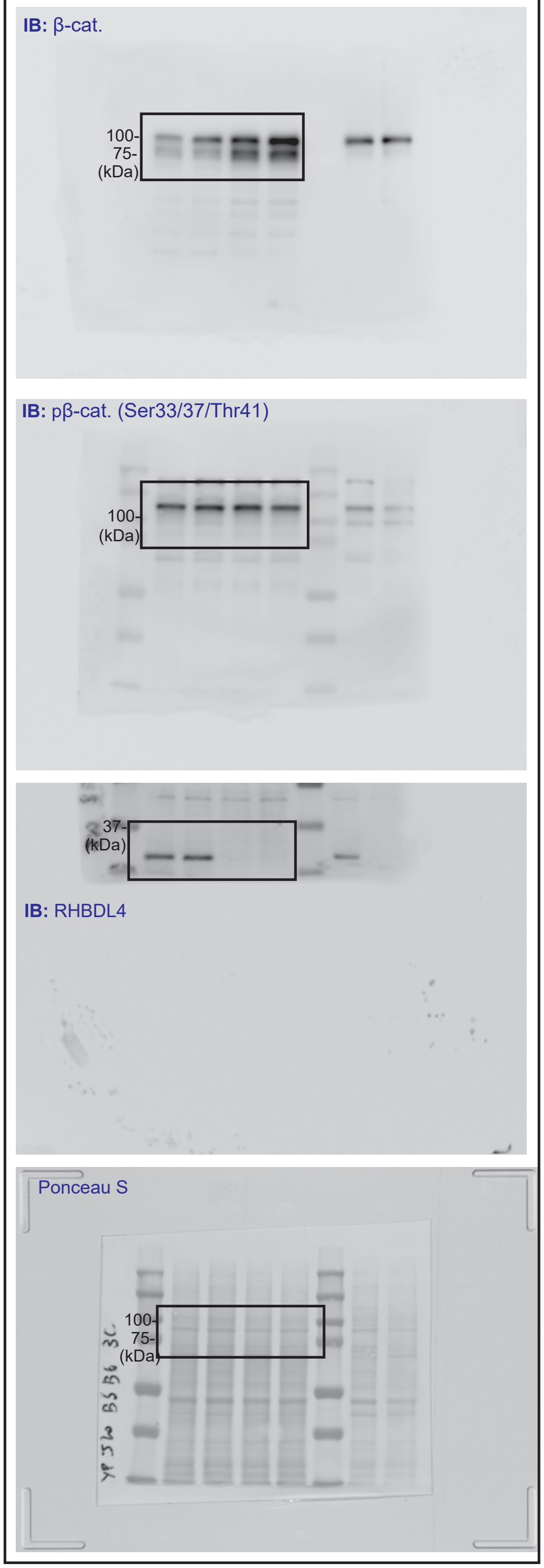

FIGURE 5C

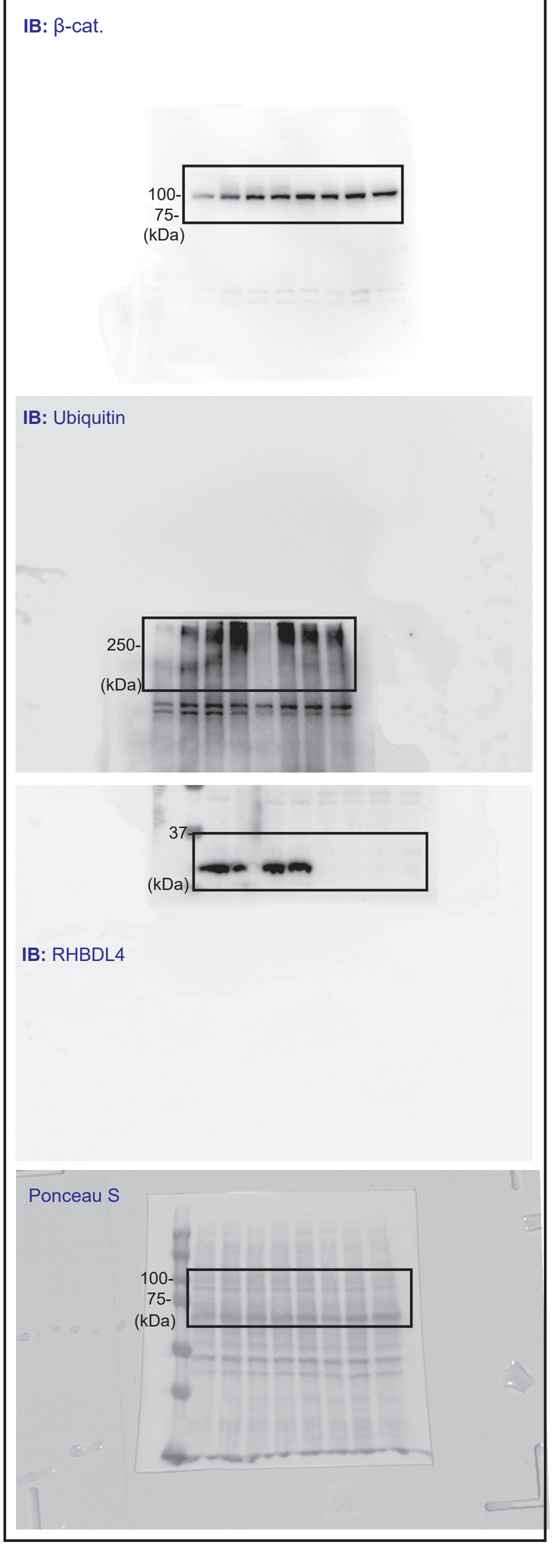

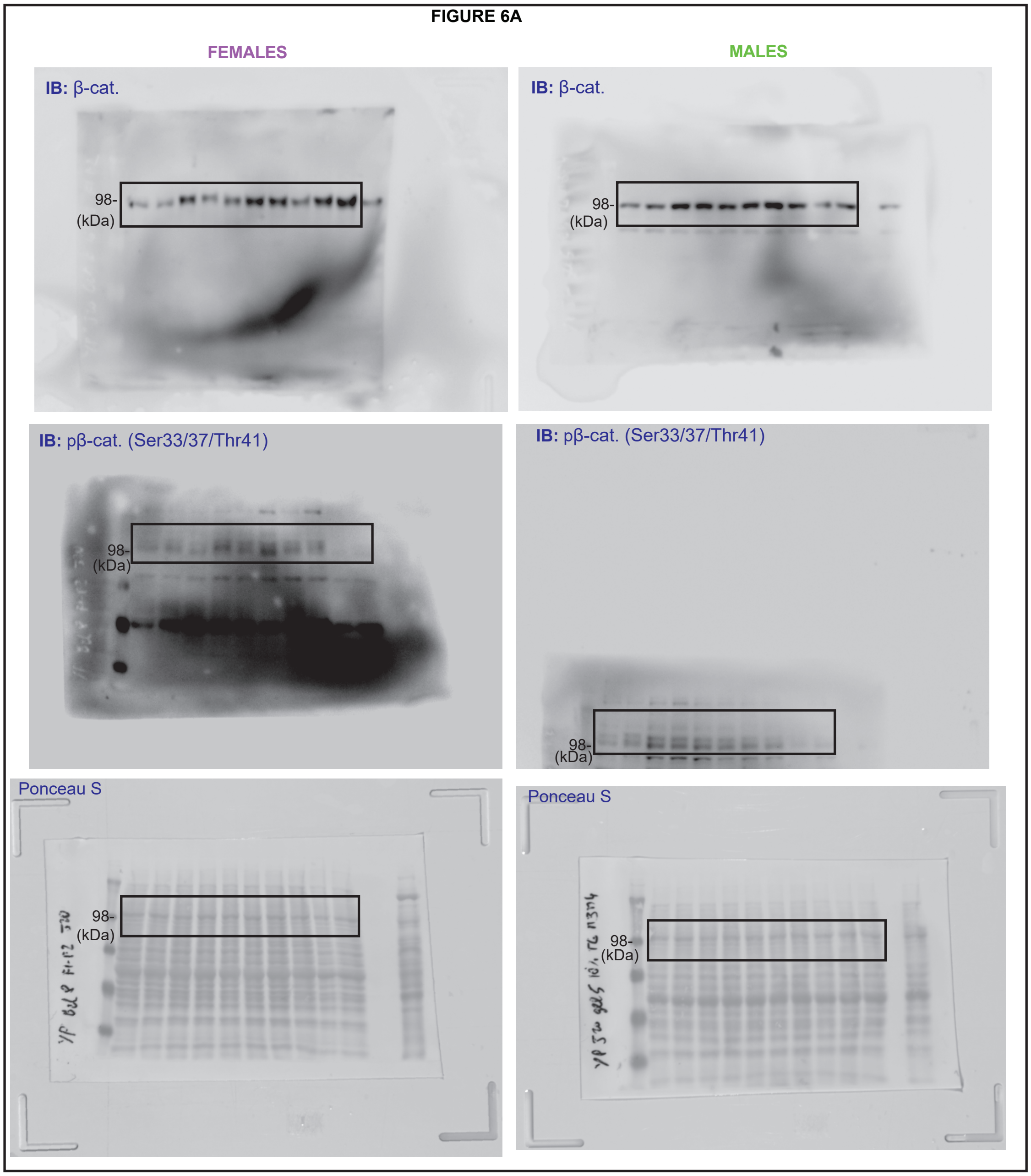
FIGURE 6A
FEMALES
MALES
IB: β-cat.
IB: β-cat.
98-
(kDa)
IB: pβ-cat. (Ser33/37/Thr41)
IB: pβ-cat. (Ser33/37/Thr41)
Ponceau S
Ponceau S
